# Soil moisture and pH differentially drive arbuscular mycorrhizal fungal composition in the riparian zone along an alpine river of Nam Co watershed

**DOI:** 10.3389/fmicb.2022.994918

**Published:** 2022-09-30

**Authors:** Yaxing Zhou, Keyu Chen, Muhammad Atif Muneer, Congcong Li, Hailan Shi, Yu Tang, Jing Zhang, Baoming Ji

**Affiliations:** ^1^School of Grassland Science, Beijing Forestry University, Beijing, China; ^2^College of Resources and Environment/International Magnesium Institute, Fujian Agriculture and Forestry University, Fuzhou, China

**Keywords:** alpine riparian soils, arbuscular mycorrhizal fungi, community distribution, driving factor, Nam Co watershed

## Abstract

The riparian zone is an important ecological corridor connecting the upstream and downstream rivers. Its highly complex biological and physical environments significantly affect the biogeographical pattern of species and various ecosystem functions. However, in alpine riparian ecosystems, the distribution patterns and drivers of arbuscular mycorrhizal (AM) fungi, a group of functionally important root-associated microorganisms, remain poorly understood. In this study, we investigated the AM fungal diversity and community composition in near-bank (wetland) and far-bank (alpine meadows) soils along the Niaqu River in the Nam Co watershed, and assessed the relative importance of abiotic and biotic filtering in shaping these distributions. Overall, 184 OTUs were identified in the riparian ecosystem, predominantly belonging to the genus *Glomus*, especially in the downstream soils, and *Claroideoglomus* in near-bank soils. AM fungal colonization, spore density, and α diversity showed an overall increasing trend along the river, while the extraradical hyphae declined dramatically from the middle of the river. AM fungal communities significantly varied between the wetland and alpine meadows in the riparian zone, mainly driven by the geographic distance, soil water content, soil pH, and plant communities. Specifically, soil pH was the principal predictor of AM fungal community in near-bank wetland soils, while soil water content had a most substantial direct effect in alpine meadows. These findings indicate that abiotic factors are the most important divers in shaping AM fungal communities at the watershed scale, which could be helpful in alpine riparian biodiversity conservation and management.

## Introduction

Arbuscular mycorrhizal (AM) fungi are widespread root-associated microorganisms and functionally important (Smith and Read, 2008). Generally, AM fungi can provide the plant with soil phosphorus and nitrogen in exchange for carbohydrates (van der Heijden et al., 2015), and improves the host plant’s resistance to drought (Gruemberg et al., 2015), heavy metals (Almeida-Rodriguez et al., 2016) and pathogens (Sikes et al., 2009; Mirshad and Puthur, 2016; Miozzi et al., 2019). There is increasing evidence that mycorrhizas are essential for regulating belowground biogeochemical cycling, soil structure, plant diversity and productivity, and ecosystem function (Van and Klironomos, 1998; Bender et al., 2015; Powell and Rillig, 2018). These mutual benefits of AM fungi are generally considered to be largely dependent on their community composition, which is mainly structured by abiotic and biotic filtering (Hempel, 2018). Therefore, the in-depth exploration of AM fungal community dynamics and their drivers is crucial if we are to manipulate these beneficial organisms to enhance ecological restoration, especially in the alpine ecosystem (Coban et al., 2022).

In recent years, with the development and application of high-throughput sequencing techniques, the development of AM fungi biogeography research has been comprehensively promoted. A meta-analysis of sequences from the global AM fungal database showed that geographic distance, soil temperature, moisture, and plant communities significantly affect the AM fungal community structure (Kivlin et al., 2011). Lekberg and Waller (2016) found that, on a global scale, plant species identity may not be more important for local AM fungal community assembly than other factors, such as environmental conditions, fungal interactions, and even random dispersal. At the landscape scale, AM fungal community composition was mainly influenced by local abiotic factors (e.g., pH, rainfall, and soil type) (Hazard et al., 2013), or land use and geographic distance (Zhu et al., 2020; Yang et al., 2021). In four typical grassland ecosystems of Sanjiangyuan National Park, we found that soil water content and plant community composition are the key factors in determining AM fungal community (Li et al., 2021a). Together, these studies suggest that both abiotic and biotic factors had strong effects on AM fungal community assembly. However, most of the related studies targeted the typical terrestrial ecosystems, and the biogeographical patterns of AM fungal communities in riparian ecosystems remain poorly understood (Li et al., 2021b).

The riparian ecosystem is an ecological transition zone between the river and the terrestrial ecosystems and provides various ecosystem services (Gonzalez et al., 2017). It is potentially a susceptible zone for interacting with biomes and environmental factors (Larsen et al., 2016). Vegetation and soils are the important components of riparian ecosystems, carrying various ecosystem service functions. For example, riparian soils intercept water resources and nutrients in rivers by regulating plant resource acquisition, soil conservation, and microbial fixation or slowly release downstream through hydrological processes, thereby forming natural nutrient and water gradients (Mitsch and Gosselink, 2000). Nevertheless, most of the current research on riparian zones has focused on the above-ground plant community composition and their distribution patterns (Acker et al., 2003; Bosompemaa et al., 2021; Qin et al., 2021). This unique natural environment also provides suitable conditions for microbial biogeography research and helps answer fundamental scientific questions in microbial ecology, such as the spatial distribution pattern of microbial communities and their maintaining mechanism. Moreover, it is also helpful for fully understanding and utilizing riparian microbial resources and further comprehending the functioning of riparian ecosystems.

Qinghai-Tibet Plateau (QTP), known as the Asian Water Tower, is dotted with numerous glaciers and lakes and is the source of large rivers in Asia (Immerzeel et al., 2010). Currently, there are 12 large watersheds on the QTP, each composed of many small watersheds with independent hydrological processes. The small watersheds with an area of 40-500 km^2^ account for about 73% of the total watershed area and about 95% of the total watershed number (unpublished data). In the ubiquitous alpine small watershed, different ecosystems (grassland, meadow, wetland, etc.) from the ice edge to the estuary are connected by the water resource and material cycling driven by the hydrological process, forming a continuous riparian ecosystem. Our previous studies found that AM fungal distribution pattern and their symbiosis with host plants are related to annual precipitation (Zhang et al., 2016, 2019). But it should be noted that the precipitation gradient of the QTP is different from that of the riparian zone within a small watershed. Besides natural precipitation, the riparian zone also includes the water flowing from rivers fed by glacial meltwater and underground water. Whenever the river flow increases during the monsoon period (Yu et al., 2019), the AM fungal propagules on the riparian zone may be dispersed into the sediments and riparian soils on the river bank or downstream by flood shocks (Harner et al., 2011), thereby affecting the distribution of AM fungi. The hydrological processes in the alpine watershed are active, and the material turnover rate is fast and continuous, so the different locations and types of ecosystems in the riparian zone are not completely independent. Therefore, the rules associated with static soil environments may not apply to riparian continuum environments (Read et al., 2015). However, AM fungal population and community dynamics in riparian along the alpine watershed are not yet explicit.

In this study, riparian soils were collected along the Niaqu river, a representative area of the small alpine watershed in QTP (Yu et al., 2019), to reveal the AM fungal distribution and the role of the local environment and geographical distance in determining community composition. To account for the vertical and horizontal dimensions of these small watersheds, samples were taken from 6 sites along the 35-km riparian zones near and away from the river, respectively. We hypothesized that; H1, AM fungal propagules, i.e., spores and extraradical hyphae, and α diversity would increase along the river from upstream to downstream, especially near-bank due to the water connectivity. H2, environmental factors, especially the heterogeneity of soil moisture caused by distance from the river, are the more important factors in structuring the fungal communities at the small watershed scale.

## Materials and methods

### Study site and sampling design

**FIGURE 1:**
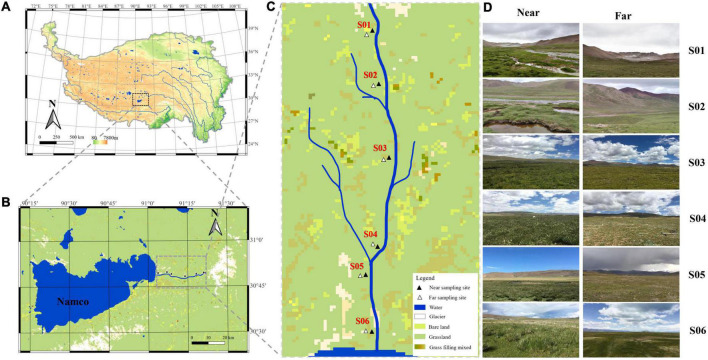
Maps showing locations of the sampling sites: **(A)** Location of Nam Co in the Qinghai-Tibet Plateau. **(B)** Map of a panoramic view of the Nam Co area marked with Niyaquriver. **(C)** Map of a magnified view of the Niyaqu basin with the six sampling sites labeled as S01–S06 along the river. **(D)** View of the sampling sites along the Niyaquriver in the Nam Co area.

Nam Co (30°30′∼30°55′N, 90°16′∼91°03′) is located at the northern foot of the Nyenchen Tanggula Mountains, with an area of 2,020 km^2^ and an average elevation of 4,718 m. It is the highest saltwater lake in the world (Anslan et al., 2020). The Nam Co basin is surrounded by high mountains and hills, forming a well-closed internal flow area. The lake water is supplied by the glacial meltwater and many small or large streams along the lake, forming many small watersheds (Zhu et al., 2010). Niaqu river is located on the east bank of the Nam Co. This area is characterized by high terrain, cold climate, and large temperature difference between day and night. A total of 12 sampling sites were set up, i.e., near-bank and far-bank along the river water flow direction (Figure 1). The vegetation types are divided into wetland (close to the river) and alpine meadows (about 250 m away from the river). The wetland is dominated by *Kobresia tibetica*, *Kobresia pygmaea*, and *Polygonum viviparum*, and the alpine meadows are dominated by *Kobresia pygmaea*, *Carex moorcroftii*, and *Leontopodium pusillum*.

By the end of August 2019, three 50 × 50 cm quadrats, with a separation distance of not less than 10 m, were randomly selected at each sample site. In each quadrat, the aboveground biomass was calculated by collecting all plant shoot and oven-dried at 80°C to a constant weight. Three soil cores (0-20 cm depth; 7 cm in diameter) were randomly collected and pooled into one sample. All the soil was passed through a 2 mm sieve *in situ*, and the fine living roots were first picked out. All samples were stored at 4°C and transported to the laboratory quickly for subsequent analysis. The living roots were thoroughly mixed and used for the determination of AM fungal colonization. The sieved soil was divided into two sub-samples (about 100 g each). The sub-sample 1 was stored at −20°C for the analysis of DNA extraction, available phosphorus, ammonium nitrogen, and nitrate nitrogen. The sub-sample 2 was air-dried and kept for later use analysis of soil physicochemical properties, such as mycelium density, spore density, pH, soil organic carbon, soil total nitrogen, and soil total phosphorus.

### Determination of soil physicochemical properties

Soil water content (SWC) was determined by drying soil samples at 105°C for 24 h. Soil pH value was detected with a soil suspension, a soil-water ratio of 1:2.5 (w/v), and a composite glass electrode (Lu, 1999). Soil organic carbon (SOC) was measured by the potassium dichromate external heating method (Nelson and Sommers, 1996). The Kjeldahl method determined total nitrogen (TN) (Bremner and Mulvaney, 1982). Ammonium nitrogen (NH_4_^+^) and nitrate-nitrogen (NO_3_^–^) were both extracted with 2 mol ⋅ L^–1^ KCl solution and then measured by flow analyzer (TRAACS 2000) (Lu, 1999). Soil total phosphorus (TP) and available phosphorus (AP) were, respectively, extracted with KClO_4_-H_2_SO_4_ and NaHCO_3_, and determined by the molybdenum-antimony colorimetric method (Bray and Kurtz, 1945; Olsen, 1954).

### Analyses of arbuscular mycorrhizal fungal colonization, spores, and extraradical hyphae

Roots were washed carefully with tap water and cut into segments of 1 cm in length. Approximately 100 root segments were randomly chosen and cleared in 10% KOH at 90 °C and stained with 0.05% Trypan blue. Roots segments were randomly selected and quantified for the percent root length of AM fungal colonization (% RLC) using the magnified intersection method at 200 × magnification (Mcgonigle et al., 1990; Brundrett et al., 1994). Spores of AM fungi were separated from 20 g of dry soil in each soil sample by wet sieving and sucrose centrifugation (Brundrett et al., 1994). The spore density was counted using a dissecting microscope. Extraradical hyphae of AM fungi in each dry soil sample were extracted by the membrane filter technique and stained with Trypan blue following the protocols of Brundrett et al. (1994) and Miller et al. (1995). Hyphal length density (HLD) (m ⋅ g^–1^ dry soil) was calculated using a line intersection method (Brundrett et al., 1994).

### DNA extraction and PCR

DNA was extracted from 0.5 g of soil samples (fresh weight) using the PowerSoil^®^ DNA Isolation Kit (MoBio Laboratories, Inc., Carlsbad, CA, USA) according to the instructions of the manufacturer. The quality and quantity of the extracted DNA were determined by electrophoresis on a 1.0% agarose gel and spectroscopic analysis (NanoDrop Technologies, Wilmington, DE, USA). DNA extracts were stored at −20°C and then 10-fold diluted DNA was used as templates of subsequent PCRs. Partial small subunit (SSU) ribosomal RNA gene fragments were amplified using nested PCR. DNA was first amplified using the primers NS31 (TTGGAGGGCAAGTCTGGTGCC) and AML2 (GAACCCAAACACTTTGGTTTCC) to amplify the 18S rRNA gene (Lumini et al., 2010). PCR was carried out in a final volume of 25 μl consisting of 2 μl extracted DNA dilution and 1 μl (10 μM) of each primer using the 2 × Taq PCR master mix system (Tiangen Biotech.) with the following cycling conditions: initial denaturation at 94°C for 3 min, followed by 30 cycles at 94°C for 30 s, 59°C for 1 min, 72°C for 2 min, and a final extension period at 72°C for 10 min. An aliquot of 2 μl of the first PCR product was diluted 1/100 with ddH_2_O and used as a template for the second PCR reaction using primers AMV4.5NF (AAGCTCGTAGTTGAATTTCG) and AMDGR (CCCAA CTATCCCTATTAATCAT) (Geel et al., 2014). The second PCR was carried out in a final volume of 50 μl with the following cycles: 94°C for 3 min, followed by 30 cycles at 94°C for 30 s, 58°C for 1 min, 72°C for 1 min, and a final extension period at 72°C for 10 min. Second-step PCR products were purified with an agarose gel DNA purification kit (AP-GX-250G; Axygen, United States) and quantified using a NanoDrop 8000. The purified products were pooled in equimolar amounts and then sequenced on the Illumina-MiSeq platform (Guangdong Magigene Inc., Guangdong, China).

### Bioinformatics analysis

The raw data obtained after high-throughput sequencing were mainly used in the Quantitative Insights Into Microbial Ecology (QIIME) and the UPARSE pipeline to analyze (Caporaso et al., 2010; Edgar, 2013). Quality control was conducted with QIIME, and high-quality sequences were imported into a Usearch (vision 11). Then, the Usearch was used for dereplication and merging of paired-end reads. The singletons were removed, and the chimeras were detected and removed using the *de novo* approach. The operational taxonomic units (OTUs) were clustered at 97% similarity. The sequence with the most occurrences in each OTU was selected as the representative sequence, and the QIIME was used for OTU classification and OTUs with non-AMF. OTUs with less than 3 samples and sequences with less than 0.01% of the total sequences were removed to reduce errors. All samples are resampled according to the minimum sample sequence size to reduce errors caused by different sample sizes. Taxonomic assignment was performed by blasting the representative sequence of each OTU against the GenBank database^1^ and the Maarj *AM* database using BLAST software. The criteria were set to >97% similarity, >90% coverage, and >200 BLAST score values. The sequences obtained in this study have been submitted to the GenBank database (PRJNA843517).

### Statistical analysis

All statistical analyses were undertaken using R^2^. Before further analysis, all data were tested for normality. Any data which did not meet normality distribution were converted using the appropriate method. The α diversity of AM fungi was characterized by calculating the OTU numbers (richness) and Shannon-Wiener index using the “diversity” function of the “Vegan” package in the R. The Kruskal-Wallis Test was used to analyze the differences in the α diversity of AM fungi, soil, and plant parameters at different locations in the riparian zone. Differences in plant community and AM fungi community composition (based on Bray-Curtis distance) were analyzed using non-metric multidimensional scaling analysis (NMDS) ordination (“Vegan” package, “metaMDS” function). Differences in community composition between groups were calculated and compared using non-parametric multivariate analysis of variance (PERMANOVA). Correlations between geographic distance, environmental and plant factors, and AM fungal communities were determined by the mantel test analysis based on the Pearson correlation. Canonical correspondence analysis (CCA) was carried out to explore the association between AM fungi community composition and soil factors using the “Vegan” package in R. Geographic distances are calculated using the “distm” function of the “geosphere” package in R. In this study, a structural equation model was constructed by Amos 25 to analyze how geographic distance, soil factors, and plant factors directly or indirectly affect AM fungal diversity and community composition. The plant and environmental variables were selected after collinearity testing based on driver analysis of AM fungal abundance, α diversity, and β diversity. Among them, geographic distance is represented by the actual longitudinal distance from the first sampling site to each of the other sites in near-bank and far-bank, respectively. Soil fertility index (SFI) was a synthetic index generated from the first principle component (PC1) scores of principal component analysis (PCA) of SOC (loading value = 0.50), TN (0.55), and TC (0.54), with 66.72% of the total explainable variance. Plant community and AM fungal community composition are characterized by the corresponding NMDS score values, respectively. The reference standard of the model fit is: chi-square/df < 2, *P* > 0.05; root mean square error of approximation (RMSEA) < 0.05; goodness of fit index (goodness of fit index, GFI) > 0.95 (Hooper et al., 2008). All graphs in this article were drawn using R and SigmaPlot 13.0.

## Results

### Soil physicochemical properties and plant characteristics

**FIGURE 2:**
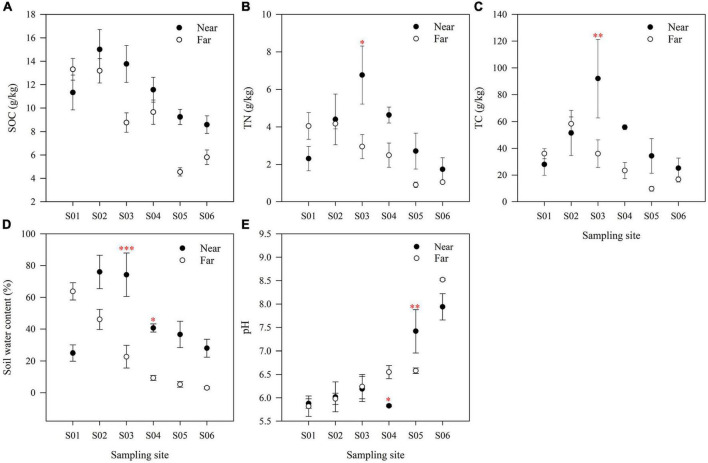
The soil organic C **(A)**, soil total N **(B)**, soil total C **(C)**, soil water content **(D)** and soil pH **(E)** at each sampling site in the riparian zone. The data are means ± SE (*n* = 3). Significant differences between the near-bank and the far-bank samples at the same sampling site were determined based on the Kruskal-Wallis Test (*P < 0.05; **P < 0.01; ***P < 0.001).

Soil physicochemical properties changed significantly along the riparian zone (Figure 2). SOC showed an overall decreasing trend along the river (Figure 2A). In contrast, soil TN (Figure 2B) and TC (Figure 2C) showed a first increasing trend and then decreasing trend in the near-bank but a continuous decreasing trend in the far-bank. Soil water content also showed decreasing trend along the river (Figure 2D). Vertical to the river, the SOC and soil water content in the near-bank were higher than those in the far-bank, except for the S01 point near the ice edge (Figures 2A,D). And except for the S01 and S02 points, the soil TN and TC in the near-bank were also higher than those in the far-bank (Figures 2B,C). The remaining soil factors, such as soil TP, AP, NH_4_^+^, and NO_3_^–^, showed no noticeable change (Supplementary Table 1).

Among the plant variables, there were no significant changes in plant aboveground biomass and richness from upstream to downstream. The aboveground biomass in the near-bank was significantly higher than that in the far-bank (*P* < 0.05), but the plant richness showed no significant difference (Supplementary Table 1). As shown on the NMDS ordination (Supplementary Figure 1), the plant species composition in the far-bank was relatively dispersed and well separated from that in the near-bank, which was verified by PERMANOVA (Supplementary Table 2).

### Identification of arbuscular mycorrhizal fungal community

**FIGURE 3:**
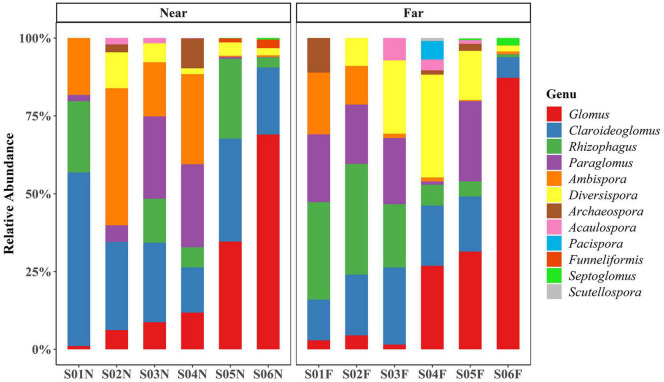
The relative abundance of each AM fungal genus detected at each sampling site.

The results of the OTU rarefaction curve indicated that the sampling intensity was sufficient (Supplementary Figure 2), which also showed that much more AM fungal sequences were detected in the far-bank than the near-bank. In this study, a total of 184 AM fungal OTUs were identified, belonging to 9 families and 12 genera (Figure 3). Among them, the most dominant genus was identified as *Glomus* (23.84% of all AM fungal sequences), mainly concentrated in the downstream riparian zone (Figure 3), followed by *Claroideoglomus* (23.32%), which mostly gathered in the near-bank.

### Arbuscular mycorrhizal fungal abundance, diversity, and community composition

**FIGURE 4:**
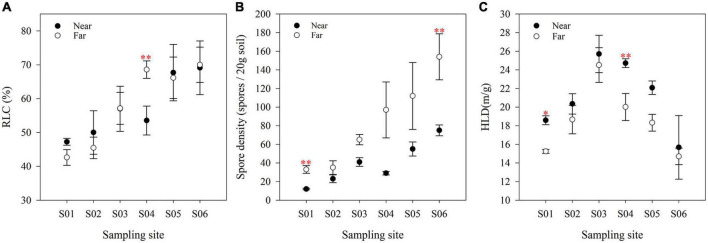
The RLC **(A)**, spore density **(B)** and HLD **(C)** of soils at various sites in the riparian zone. The data are means ± SE (*n* = 3). Significant differences between the near-bank and the far-bank samples at the same sampling site were determined based on the Kruskal-Wallis Test (*P < 0.05; **P < 0.01; ***P < 0.001).

AM fungal abundance was significantly different along the river and showed contrasting patterns (Figure 4). The RLC (Figure 4A) and spore density (Figure 4B) showed an overall upward trend along the river, while the HLD (Figure 4C) first increased and then decreased, reaching a peak at point S03. Vertical to the river, the colonization of AM fungi was not significantly different between the near- and far-bank soils. The spore density in the far-bank was much higher than that in the near-bank soil (*P* < 0.05), while the HLD showed a reverse trend (*P* < 0.05; Supplementary Table 3).

**FIGURE 5:**
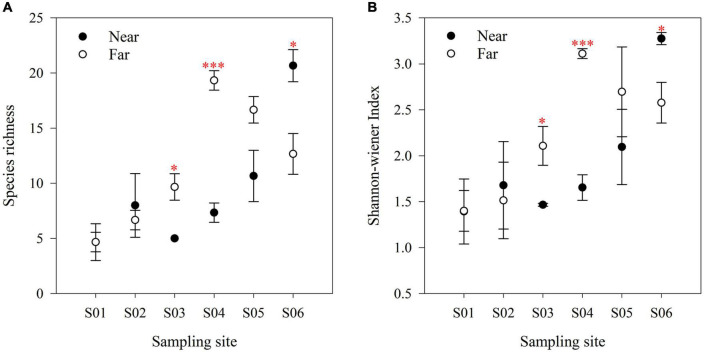
AM fungal species richness **(A)** and Shannon-wiener index **(B)** in riparian zone. The data are means ± SE (*n* = 3). Significant differences between the near-bank and the far-bank samples at the same sampling site were determined based on the Kruskal-Wallis Test (*P < 0.05; **P < 0.01; ***P < 0.001).

**FIGURE 6:**
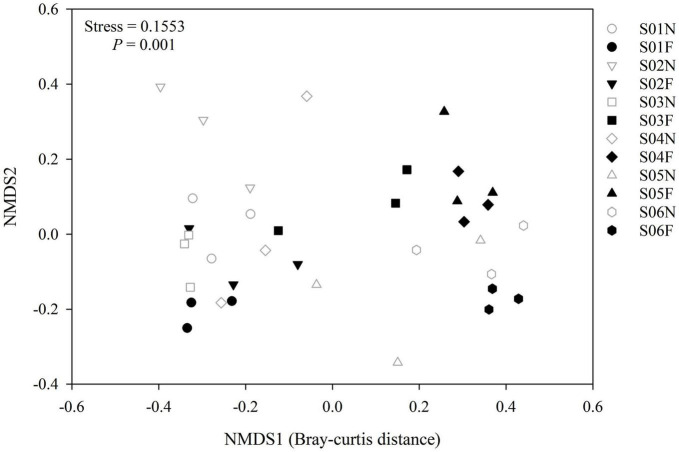
Non-metric multidimensional scaling (NMDS) ranking according to the AM fungal community differences (Bray-Curtis distance) identified at each sampling site. Permutational multivariate analysis of variance (PERMANOVA) was adopted to compare community composition among different soil samples.

In the near-bank soils, the AM fungal α diversity (species richness and Shannon-wiener index) increased overall from upstream to downstream along the river (Figure 5), and there was a significant difference in the community composition (*P* = 0.001; Figure 6). While in the far-bank soils, AM fungal community also showed a distinct distribution pattern along the river (*P* < 0.05; Figure 5), but its α diversity increased and then decreased at the two sampling sites near the lake (Figure 5). On the whole, the α diversity in the near-bank was not significantly different from that in the far-bank, except for the specific S03, S04, and S06 (Supplementary Table 4).

### Drivers of arbuscular mycorrhizal fungal diversity and community composition

**FIGURE 7:**
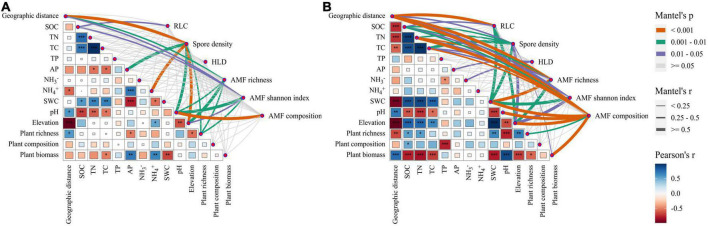
Relationships between AM fungal abundance, α diversity, and community composition with environmental factors in the near **(A)** and far **(B)** bank soils. The relationships among environmental variables were illustrated by Pearson correlation (*P < 0.05; **P < 0.01; ***P < 0.001).

The results of the Mantel test showed that a variety of environmental factors such as geographic distance, plant community, and soil physicochemical were significantly correlated with AM fungal abundance, α diversity, and community composition (*P* < 0.05, Figure 7). In the near-bank soils, the AM fungal spore density significantly correlated with geographic distance, elevation, soil pH, NH_4_^+^ content, and AP content, respectively (Figure 7A). The α diversity showed significant correlations with soil pH, elevation, plant richness, and geographic distance. In contrast, AM fungal community composition was only significantly correlated with soil pH. In the far-bank soils, AM fungi were influenced by more complex biotic, and abiotic factors (Figure 7B). Geographic distance, soil water content and elevation significantly correlated with AM fungal communities. Moreover, the output of the Canonical correspondence analysis (CCA) with environmental factors indicated that the AM fungal community composition was significantly impacted by soil pH (*P* < 0.001), geographic distance (*P* < 0.001), and soil water content (*P* < 0.05; Supplementary Figure 3). It is worth noting that a valid (*P* < 0.001) distance-decay relationship was observed for the AM fungal community over geographic distance with similar slopes (turnover rates) in the near (slope = -0.017, R^2^ = 0.150) and far (slope = −0.015, R^2^ = 0.369) river-bank soils (Supplementary Figure 4).

**FIGURE 8:**
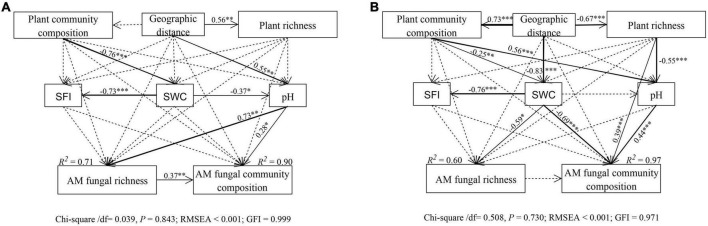
Structural equation model (SEM) showing causal relationships among geographic distances, plant variables, soil variables, AM fungal richness and community composition in the near **(A)** and far **(B)** bank soils. Solid and dashed lines indicate significant and non-significant pathways, respectively. The width of the solid line indicates the strength of the causal effect. The R^2^ value represents the proportion of variance explained for each variable.

The structural equation model (SEM) further better assessed the impact of environmental variables on AM fungal community composition through direct and indirect pathways. In total, AM fungal species richness and community composition in the riparian soils were mainly driven by geographic distance and soil water content, followed by soil pH and plant community composition (Supplementary Figure 5 and Supplementary Table 5). Among them, the SEM model (Chi_square/df = 0.039, *P* = 0.843, RMSEA < 0.001; GFI = 0.999) explained 71 and 90% of the AM fungal richness and community composition variance in the near-bank soils, respectively (Figure 8A and Table 1). In this model, soil pH had direct effects on AM fungal species richness (standard estimates = 0.73, *P* < 0.01) and community composition (standard estimates = 0.28, *P* < 0.05), while geographic distance and soil water content had indirect effects on AM fungi via changes in soil pH. The far-bank SEM model (Chi_square/df = 0.508, *P* = 0.730, RMSEA < 0.001; GFI = 0.971) explained 60 and 97% of the AMF richness and community composition variance, respectively (Figure 8B and Table 2). In the far-bank soil, other than the direct effect of soil water content, geographic distance indirectly affected AM fungal species richness and community composition through soil moisture (*P* < 0.05). Soil pH showed a significant direct effect on AM fungal community composition. Moreover, plant community composition also significantly affected AM fungal community composition in the total riparian zone.

**TABLE 1:** Standardized total contribution rate of main factors driving AM fungal richness and community composition in the near-bank soils.

Explanatory factor	Standardized Total Effects	
	AM fungal richness	AM fungal community composition
Geographic distance	0.504	0.567
Plant community composition	0.086	0.436
Plant richness	0.276	0.191
SWC	–0.573	–0.568
pH	0.726	0.551
SFI	–0.071	0.140

**TABLE 2:** Standardized total contribution rate of main factors driving AM fungal richness and community composition in the far-bank soils.

Explanatory factor	Standardized Total Effects	
	AM fungal richness	AM fungal community composition
Geographic distance	0.698	0.876
Plant community composition	0.179	0.413
Plant richness	0.305	0.249
SWC	–0.689	–0.934
pH	–0.077	0.434
SFI	0.182	–0.035

## Discussion

### Arbuscular mycorrhizal fungal abundance and diversity in the riparian zone of small alpine watershed

Previous studies have shown that arbuscular mycorrhizal symbionts commonly exist in extreme alpine stress habitats on the Qinghai-Tibet Plateau (Gai et al., 2009; Li et al., 2018) and play an essential role in typical alpine grassland plants growth and community succession (Jiang et al., 2018; Mao et al., 2019). Our study also proved that a relatively high AM fungal abundance was present in the riparian zone of the alpine watershed. The dominant genera *Glomus* and *Claroideoglomus* are common AM fungi groups in typical alpine grassland (Shi et al., 2017; Jiang et al., 2018; Bahaduiv et al., 2019). Moreover, we found that the relative abundance of *Glomus* significantly increased along the river while the *Claroideoglomus* was the dominant genus in the near-bank soils. Fungi in the *Glomus* are considered to be more disturbance-tolerant due to their “r” selected traits, which can produce a large number of spores and mycelium in the process of growth and reproduction, thus enhancing their adaptive ability (Ohsowski et al., 2014; van der Heyde et al., 2017b). Therefore, the potential reasons for the higher abundance of *Glomus* in the downstream riparian zone are as follows. First, the subsurface flow in the riparian zone will enhance the accumulation of the *Glomus* spores with a small diameter and large production. Second, the frequent wildlife and livestock activities downstream near the lake are also conducive to promoting the predominance of the *Glomus*, which are considered more disturbance tolerant (Ba et al., 2012; van der Heyde et al., 2017a,b). While the *Claroideoglomus* usually showed higher salt tolerance and predominated in high saline soils (Dai et al., 2013; Dashtebani et al., 2014; Analia Silvani et al., 2017; Yang et al., 2020). Influenced by the carbonate minerals of the river water (Gao et al., 2008; Keil et al., 2010), the soil salinity in the near-bank may be relatively high than that in the far-bank soils, which might be why the relative abundance of *Claroideoglomus* was much higher in near-bank soil. Thus, further research is needed to carry out to test the relative importance of soil salinity for AM fungal assembly in the riparian zone.

It is worth noting that the same high AM colonization levels observed in wetland plants (near-bank) and alpine meadow plants (far-bank) were inconsistent with other previous work, which showed that AM fungal colonization is lower in wetland-aquatic habitats than in terrestrial ecosystems (Fusconi and Mucciarelli, 2018). On the one hand, there is no significant difference in plant species diversity between the wetland and alpine meadow in this study, which might provide equally rich ecological niches for AM fungi. On the other hand, the near-river alpine wetlands, usually classified as wet meadows, are just inundated with river water temporarily during the growing season in this area. The effect of flooding intensities on AM colonization is also weaker than that of aquatic habitats, which is featured in typical oxygen limitations unsuitable for AM fungal survival (Bohrer et al., 2004). However, the higher soil moisture near-bank did affect the mycorrhizal growth and development, as confirmed by the lower spore density and α diversity. Conversely, the higher aboveground biomass meaning more allocation of carbon to AM fungi in the near-bank may result in increased external hyphae, which concurs with our findings that HLD in the near-bank soils was much higher than that in the far-bank.

This study demonstrated that AM fungal spores and α diversity along the Niaqu river showed an overall increasing trend from upstream to downstream, which supports our first hypothesis (H1) and corroborates with a recent study showing the increased microbial abundance and diversity along the Nu River (Zhang et al., 2022). The potential reasons for these results may be due to the great elevation drop from upstream to downstream (about 400 m), which might enhance the accumulation of the microbial propagules under the transportation of subsurface flow and increase the AM fungal community α diversity. Furthermore, a large amount of the *Glomus* group gathered downstream might provide many asexual spores (Chagnon et al., 2013). At last, the gradually favorable soil moisture and pH from upstream to downstream might support multiple AM fungal phylotypes colonization (Deepika and Kothamasi, 2015), which can be confirmed by the strong correlations between soil water content and soil pH and AM fungi.

### Different major driving factors on arbuscular mycorrhizal fungal community between the wetland and alpine meadows in the riparian zone

Exploring the driving factors of AM fungal community composition in grassland ecosystems is one of the primary aims of mycorrhizal ecology (Ma et al., 2021). Previous studies have shown that the main factors influencing the grassland AM fungal community composition included host plants, geographical, climatic, and soil characteristics (Davison et al., 2015). Still, the relative importance of these driving factors is conflicting, especially at different scales (Hempel, 2018). Our results proved a strong effect of geographic distance on AM fungal diversity and community composition at the alpine small watershed scale. This is in line with previous studies reporting that potential spatial constraints may be a significant determinant of AM fungal community formation even at tiny scales (Hazard et al., 2013; Horn et al., 2014, 2017). However, the spatial isolation would be somewhat weakened by the water connectivity, especially in near-bank soils, thus showing a weaker distance-decay relationship. Notably, geographic distance only indirectly affected AM fungi through soil pH in near-bank and soil water content in far-bank, respectively. These findings suggest that local abiotic environmental variation caused by spatial differences is more critical in shaping AM fungal community in the Niaqu small watershed, supporting our second hypothesis (H2).

The effects of soil water content and pH on the microbial community have been widely reported, which can vitally shape the distribution pattern of soil microbes (Liu et al., 2018; Neupane et al., 2022). In this study, we also found the dominant role of these two factors in AM fungal distribution in the alpine riparian ecosystems. However, it is worth noting that in the near-bank soils, soil pH rather than soil water content exerted a substantial direct effect on AM fungal species richness and compositions, contrary to our second hypothesis. But in the far-bank soils, our results indicated soil moisture to be the strongest predictor of AM fungal community compositions. We assumed that the relative importance of these two drivers in determining the observed fungal distribution patterns mainly depends on the limiting factors of habitats in the riparian zone.

Unlike the near-bank wetland, where the soil is nearly saturated, soil moisture is likely to be the limiting factor of the typical alpine meadow ecosystem in the far-bank. Especially the sharp declined soil water content from upstream to downstream could influence AM fungal species directly through water limitation (Fu et al., 2022). Soil moisture can directly affect AM fungal colonization (Fitzsimons et al., 2008), the growth and distribution of extra-radical mycelium (Clark et al., 2009), and the specific species assemblages (Staddon et al., 2004; Deepika and Kothamasi, 2015). At the same time, soil aeration can influence fungal growth and diversity due to their high oxygen requirement (Miller and Sharitz, 2000). Although the soil water content also showed a decreasing trend from upstream to downstream in the near-bank wetland, the overall high soil moisture level might reduce the community sensitivity of AM fungi to soil water change (Garcia et al., 2008). Here, we found that soil pH, even within a limited range of about 2.0 pH units, was the main direct factor controlling the fungal community distribution in the near-bank soils. Moreover, soil moisture also indirectly affected the AM fungal community via changes in the soil pH. Soil pH is likely to affect the arbuscular mycorrhizal sporulation, extraradical mycelium growth, and the niche space, especially for specific genera (Van Aarle et al., 2002; Dumbrell et al., 2010). For instance, the relative abundance of the *Glomus* was shown to increase with increased soil pH along the river, either in the near or far-bank soils in this study.

Host plants are essential in regulating AM fungal structure and community (Neuenkamp et al., 2018). However, in this study, we did not find a strong effect of host plants on AM fungal community compared to abiotic factors, consistent with the results by Yang et al. (2021) which reported a minor contribution of crop types. This may be due to the specific plant types in this watershed ecosystem. The near-bank wetland and far-bank alpine meadows are dominated by the Cyperaceae plants, which do not strongly depend on AM fungi (Newman and Reddell, 1987; Fuchs and Haselwandter, 2004). Thus the biotic filtering is relatively unimportant in driving AM fungal community composition compared to abiotic parameters in this area. Similarly, very little AM fungal community variation was explained by soil fertility, mainly regulated by soil water content both in the near- and far-bank soil, confirming the driving effect of soil moisture in this watershed ecosystem.

## Conclusion

This is the first study investigating the composition and distribution of AM fungal community in the riparian zone of the alpine river on the QTP. By studying the biotic and abiotic factors related to riparian soil, we undertook an extensive assessment of the factors driving AM fungal communities. Our study reveals that AM fungal communities significantly varied between the wetland and alpine meadows in the riparian zone, mainly driven by the geographic distance, soil water content, soil pH, and plant communities. Especially, soil pH was the principal predictor of AM fungal community in near-bank wetland soils, while soil water content had a most substantial direct effect in alpine meadows. In addition, we also found that geographic distance strongly influences AM fungal community at the alpine small watershed scale, but indirectly through soil water content or pH. In contrast, host plants have minimal influence on it. Our results indicate that the abiotic factors are the most important drivers in shaping AM fungal communities at the watershed scale. The results of this study lay a foundation for fully understanding and utilizing riparian microbial resources, further comprehending the service functions of riparian ecosystems, and providing theoretical support for the construction of microbial biogeographic models.

## Data availability statement

The datasets presented in this study can be found in online repositories. The names of the repository/repositories and accession number(s) can be found below: https://www.ncbi.nlm.nih.gov/genbank/, PRJNA843517.

## Author contributions

YZ, JZ, and BJ designed the experiments, which were then carried out by YZ, and CL. YZ, KC, HS, and YT collected the data and conducted the statistical analyses. YZ wrote the first draft of the manuscript. JZ, MM, KC, BJ, and YZ reviewed and edited the manuscript before submission. All authors made substantial contributions to the discussion of content.

## Footnotes

1
http://blast.ncbi.nlm.nih.gov/

2
https://www.r-project.org/
