# Supplementary material for: Soil moisture and pH differentially drive arbuscular mycorrhizal fungal composition in the riparian zone along an alpine river of Nam Co watershed
Source: Front Microbiol. 2022 Sep 30;13:994918. doi: 10.3389/fmicb.2022.994918 (PMC9561679; doi:10.3389/fmicb.2022.994918)
Supplement: Supplementary file 1 [file Data_Sheet_1.docx]

Supplementary Material

# Supplementary Figures and Tables

## Supplementary Figures


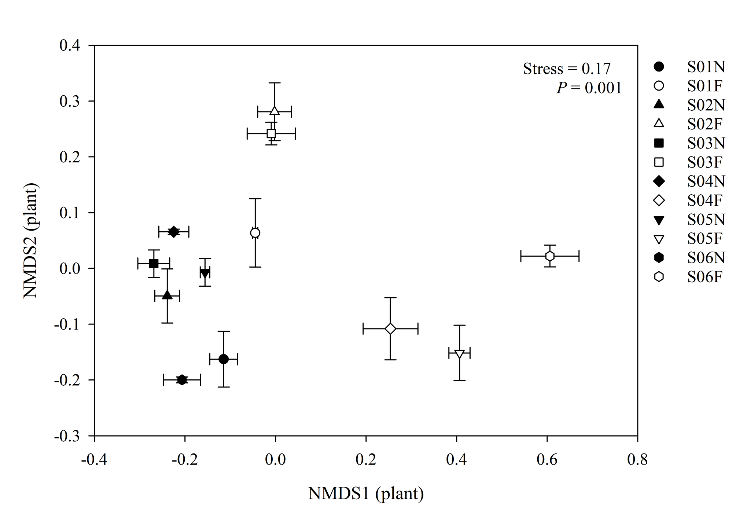


**Figure S1.** Non-metric multidimensional scaling (NMDS) ordination of plant community dissimilarities (Bray-Curtis) in different sampling site with the observed species composition. Permutational multivariate analysis of variance (PERMANOVA) was adopted to compare community composition among different sites.


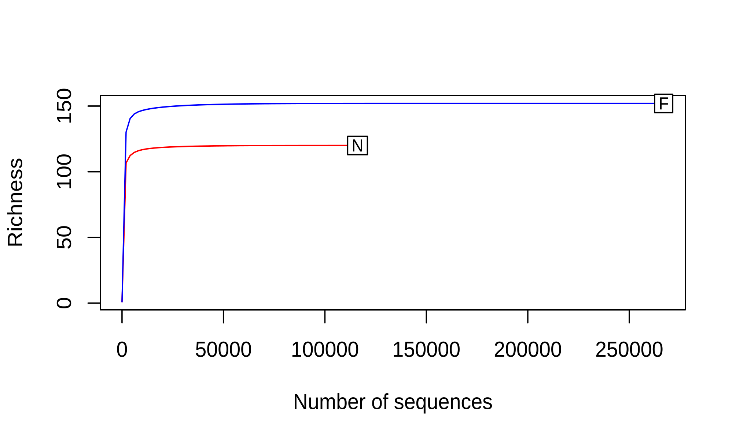


**Figure S2.** Rarefaction curve of AM fungal OTUs detected in the near- and far-bank soils.


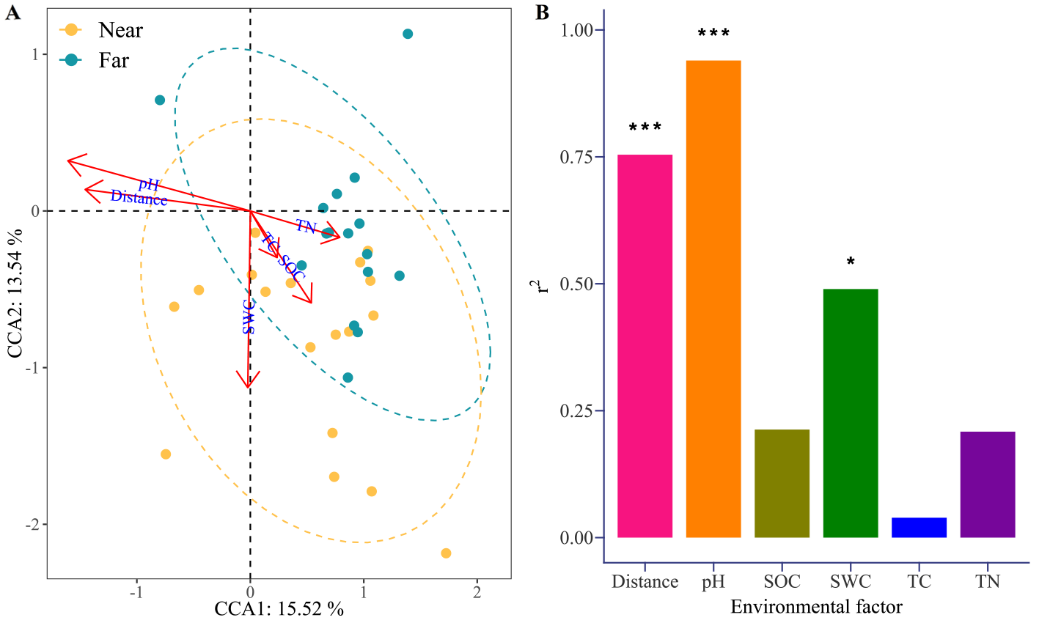


**Figure S3.** Canonical correspondence analysis (CCA) of the soil AM fungal community structure and environmental factors in the near- and far-bank (A) and the importance of each environmental factor (B). Environmental factors indicated in the red text include TN, TC, SOC, SWC, geographic distance (Distance), and pH. Significant differences between the environmental factors were determined based on the Permutation Test (**P* < 0. 05; ***P* < 0.01; ****P* < 0.001).


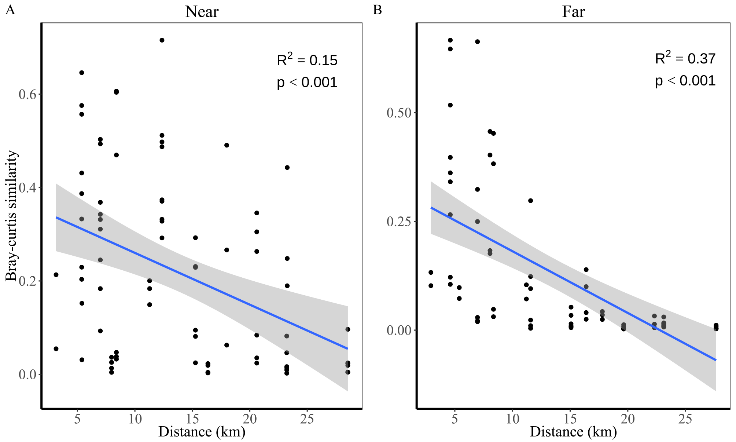


**Figure S4.** Distance decay patterns of AM fungal community composition in the near (A) and far (B) bank soils based on Bray-Curtis similarity.


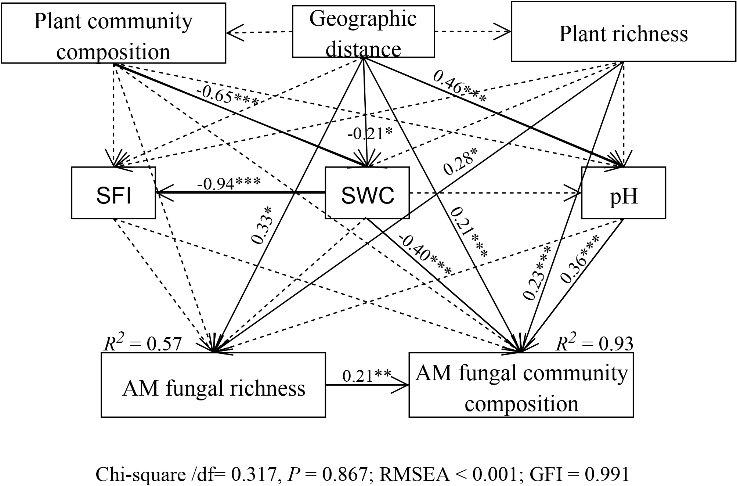


**Figure S5.** Structural equation model (SEM) showing causal relationships for geographic distance, plant variables, soil variables, AM fungal richness and community composition in the riparian zone. Solid and dashed lines indicate significant and non-significant pathways, respectively. The width of the solid line indicates the strength of the causal effect. The R^2^ value represents the proportion of variance explained for each variable.

## Supplementary Tables

**Table S1.** Differences in environmental and plant variables between the near- and far-bank soils. Data are mean ± SE (n=18). Significant differences were determined based on the Kruskal-Wallis Test (**P* < 0. 05; ***P* < 0.01; ****P* < 0.001).

| Variables | Near-bank | Far-bank |
| --- | --- | --- |
| Soil organic matter (g/kg) | 19.97±5.12 | 15.88±6.04 |
| Soil total N (g/kg) | 3.76±2.17 | 2.60±1.41 |
| Soil total C (g/kg) | 47.80±28.28***** | 30.02±17.35 |
| Soil total P (g/kg) | 0.76±0.13 | 0.74±0.17 |
| Soil available P (mg/kg) | 6.27±1.69 | 6.41±0.86 |
| Soil NO_3_^-^ (mg/kg) | 6.83±0.96 | 6.30±1.22 |
| Soil NH_4_^+^ (mg/kg) | 44.85±11.23 | 46.76±11.47 |
| Soil water content (%) | 46.70±24.21****** | 25.00±23.58 |
| Soil pH | 6.55±0.88 | 6.62±0.93 |
| Plant richness | 10.22±1.80 | 9.72±4.18 |
| Plant biomass (g/m^2^) | 28.09±11.58***** | 21.61±14.48 |

**Table S2.** PERMANNOVA analysis of plant community composition among different habitats.

| Sampling site | *R^2^* | *P_adj_* |
| --- | --- | --- |
| All | 0.71 | 0.001 |
| Near/Far | 0.21 | 0.001 |
| Near | 0.64 | 0.001 |
| Far | 0.63 | 0.001 |

**Table S3.** AM fungal abundance inside and outside the root in the near- and far-bank. Data are mean ± SE (n=18). Significant differences were determined based on the Kruskal-Wallis Test (**P* < 0. 05; ***P* < 0.01; ****P* < 0.001).

|  | RLC(%) | AC(%) | VC(%) | HC(%) | HLD  （m·g^-1^ soil） | Spore density  (spores 20 g^-1^ soil) |
| --- | --- | --- | --- | --- | --- | --- |
| Near | 57.62±12.91 | 2.10±2.16 | 16.93±4.51 | 38.59±9.58 | 22.19±5.06* | 36.67±21.28 |
| Far | 58.49±13.92 | 3.27±2.59 | 15.22±3.44 | 40.00±9.67 | 17.58±3.86 | 81.33±55.83** |

RLC, percentage of root length colonized by AM fungi; AC, percentage of root length colonized by arbuscules; VC, percentage of root length colonized by vesicles; HC, percentage of root length colonized by hyphae; HLD, hyphal length.

**Table S4.** AM fungal α- diversity in the near- and far-bank soils. Data are mean ± SE (n=18). Significant differences were determined based on the Kruskal-Wallis Test (**P* < 0. 05; ***P* < 0.01; ****P* < 0.001).

|  | richness | Shannon-Wiener index |
| --- | --- | --- |
| Near | 9.39±6.17 | 1.93±0.77 |
| Far | 11.61±6.02 | 2.23±0.78 |

**Table S5.** The standardized total effects of the main factors driving AM fungal richness and community composition in the riparian zone.

| Explanatory factor | Standardized Total Effects | |
| --- | --- | --- |
|  | AM fungal richness | AM fungal community composition |
| Geographic distance | 0.592 | 0.695 |
| Plant community composition | 0.300 | 0.516 |
| Plant richness | 0.174 | 0.154 |
| SWC | -0.471 | -0.579 |
| pH | -0.310 | 0.424 |
| SFI | 0.108 | 0.023 |
